# Control of *Erigeron bonariensis* with *Thymbra capitata*, *Mentha piperita*, *Eucalyptus camaldulensis*, and *Santolina chamaecyparissus* Essential Oils

**DOI:** 10.3390/molecules25030562

**Published:** 2020-01-28

**Authors:** Mercedes Verdeguer, Luis Guillermo Castañeda, Natalia Torres-Pagan, Juan Antonio Llorens-Molina, Alessandra Carrubba

**Affiliations:** 1 Instituto Agroforestal Mediterráneo, Universitat Politècnica de València, Camino de Vera s/n, 46022 Valencia, Spain; luicasag@posgrado.upv.es (L.G.C.); natorpa@upv.es (N.T.-P.); juallom2@qim.upv.es (J.A.L.-M.); 2 Università degli Studi di Palermo, Dipartimento di Scienze Agrarie, Alimentari e Forestali, Viale delle Scienze, Ed. 4, 90128 Palermo, Italy; alessandra.carrubba@unipa.it

**Keywords:** weed control, natural herbicides, essential oils, *Erigeron bonariensis*, pre-emergence, post-emergence, seed germination

## Abstract

In the search of sustainable and environmentally friendly methods for weed control, there is increasing interest in essential oils (EOs) as an approach to reduce synthetic herbicide use. The phytotoxicity of *Thymbra capitata*, *Mentha* *piperita*, *Eucalyptus camaldulensis,* and *Santolina chamaecyparissus* EOs against the noxious weed *Erigeron bonariensis* were evaluated in pre- and post-emergence assays in greenhouse conditions. The EOs were applied at 2, 4, and 8 µL/mL, with Fitoil used as emulsifier. In post-emergence, two ways of application were tested, irrigation and spraying. Several germination parameters (germination %, mean germination time, and synchrony of the germination process) were evaluated in pre-emergence tests, and the phytotoxicity level was assessed in post-emergence. In pre-emergence, all EOs significantly reduced seed germination as compared to the controls, ranking: *T. capitata > E. camaldulensis > S. chamaecyparissus > M. piperita*. The effectiveness of all EOs varied with the tested dose, always following the rank 2 μL < 4 μL < 8 μL, with *T. capitata* EO showing full effectiveness even at the lowest dose. In post-emergence, *T. capitata* was the most effective EO, inducing a rather complete inhibition of plantlet growth at the highest two doses. These EOs demonstrated to have good potential for the formulation of natural herbicides.

## 1. Introduction

The world population is increasing continuously and is expected to reach 9.7 billion in 2050 and 11.1 billion in 2100 [1]. Furthermore, the size of arable land is limited and cannot be expanded without threatening vital natural habitats like the rain forests [2]. Other resources necessary for agricultural production, like water, energy, biodiversity, human labor, and fertilizers, are reducing as well [3]. The challenge to feed more people in this context is to increase agricultural production and food availability in a sustainable way. One key opportunity to reach this objective is to improve crop protection. Direct yield losses caused by pest (insects, pathogens, and weeds) range approximately between 20 and 40% of global agricultural productivity [4], but these numbers do not reflect the real cost that includes indirect losses caused to consumers, public health, societies, environments, economic fabrics, and farmers [3]. Overall, weeds decrease major crop potential yields up to 34%, causing greater losses than diseases and pests [4]. Weeds reduce the quantity and quality of crop yields due to the competition for water, nutrients, light, and carbon dioxide, as well as by allelopathy [5]. 

There are different strategies for weed management, from cultural practices, like crop rotation or the use of more competitive crop varieties, to physical, mechanical, and biological control methods. However, since the discovery of the first herbicide, 2,4-dichlorophenoxyacetic acid (2,4-D), in 1940 [6], chemical control (herbicides application) has been the most used method to control weeds. The adoption of herbicides has contributed to the improvement of weed control [7], with a subsequent increase in crop yields, and has also led to a reduction of tillage operations, with a decrease in CO_2_ emissions associated with farming activities. However, their overuse promoted the development of resistant weeds, also affecting human health and causing environmental pollution [8]. 

All the aforementioned has led to the adoption of “Integrated Pest Management” (IPM) strategies, combining the different available control methods and promoting the search for alternatives to pesticides. Worldwide legislation has adopted the principles of IPM, such as the globally accepted International Code of Conduct on the Distribution and Use of Pesticides [9], the European Union Directive 2009/128/EC, or the US Food Quality Protection Act (FQPA) [10].

Natural products are an excellent alternative to synthetic pesticides as means to reduce negative impacts to human health and the environment. The need for developing new crop protection tools with novel modes of action makes the discovery and commercialization of natural products as green pesticides an attractive and profitable pursuit that is commanding attention [11]. 

Herbicides based on plant essential oils (EOs) have been demonstrated to be effective against a wide range of weeds and are a potential alternative to non-selective herbicides [12]. EOs from *Thymus vulgaris*, *Satureja hortensis*, *Cinnamomum zeylanicum*, and *Syzygium aromaticum* were phytotoxic and caused cell death to *Chenopodium album*, *Ambrosia artemisiifolia*, and *Sorghum halepense* [13]. Many studies have proved the herbicidal activity of *Eucalyptus spp*. EOs, for example, *Eucalyptus camaldulensis* EO, inhibited the germination and growth of *Amaranthus hybridus* and *Portulaca oleracea* [14], and the EO from *E. citriodora* was effective to control the invasive noxious weed *Parthenium hysterophorus* [15]. EOs from Lamiaceae and Asteraceae have also shown high potential for their use as natural herbicides [12,16,17].

*Erigeron bonariensis* L. (syn: *Conyza bonariensis* (L.) Cronquist, Asteraceae) has become one of the most troublesome weeds around the world due to its widespread occurrence, pronounced interference, and challenging management [18]. It is native to South America [19] and has invaded a large number of countries in Africa, Asia-Pacific, and Europe [20]. 

Some of the features that explain the colonizing success and high competitiveness of *E. bonariensis* are that it is an annual prolific species, capable of producing 200,000 viable seeds per plant and establishing under different environmental conditions [21]. The base, optimum, and maximum temperatures for seed germination were estimated at 4.2, 20, and 35 °C [22]. The seeds show a dynamic ecology, which enables *E. bonariensis* to reproduce and proliferate in a wide range of temperatures, moistures, and soils in different cropping systems [23]. *E. bonariensis* adapts well to systems considered soil conservationists, such as tillage, minimum tillage, and orchard areas [24,25]. The factors that can contribute to its ecological adaptability, the survival of resistant biotypes, and the high infestations in conservation systems are self-pollination, along with a large production of easily dispersible seeds [26]. It has developed resistance to glyphosate (EPSP synthase inhibitor, group D), the most commercialized herbicide in the world today [21], as well as to chlorsulfuron (ALS inhibitors, group B), atrazine, and simazine (Photosystem II inhibitors, group C1), diquat, and paraquat (PSI Electron Diverter, group D) [27]. Many agricultural producers are facing problems to control this weed in different crops around the world [20,21,22,23,24,25,26].

The objective of this research was to study the phytotoxic potential of *Thymbra capitata*, *Mentha piperita*, *Eucalyptus camaldulensis*, and *Santolina chamaecyparissus* EOs against *E. bonariensis* with the purpose to find alternatives for the control of this problematic weed. These EOs have not been tested before on this weed.

The selection of the EOs was based on the previous knowledge of their herbicidal properties. *T. capitata* EO has been reported to inhibit the germination and growth of many weeds [28,29], including *P. oleracea* and *Erigeron canadensis* [30]. *M. piperita* EO showed phytotoxic activity against tomato crops (*Lycopersicon esculentum*), radish (*Raphanus sativus*) and the weeds *P. oleracea*, *Convolvolus arvensis*, and *Echinochloa colonum* [31]. The potential of *E. camaldulensis* EO to control different weeds has been demonstrated [14,30]. The EO from *S. chamaecyparissus* could be a promising alternative to synthetic herbicides as it was tested on seed germination and root and shoot growth of four crops (*Zea mays*, *Triticum durum*, *Pisum sativum*, and *Lactuca sativa*) and two weeds (*P. oleracea* and *Vicia sativa*) resulting to be less injurious to the crop species *Z. mays* and *P. sativum* as compared to the weeds [32]. 

## 2. Results and Discussion

### 2.1. Pre-Emergence Treatments

The results of the statistical analysis carried out on all germination measurements in the pre-emergence treatments (four EOs and two controls: C1, water and C2, water + Fitoil) are reported in Table 1. After 40 days of observations, the total germination percentage value reached 28% in C1, whereas in C2 seed germination was almost twice as high (54%). The highest values of seed germination were recorded in the two controls, above all C2, that apparently exerted a stimulation effect.

The strongest inhibitory effect was recorded in the pots treated with EOs of *T. capitata*, *E. camaldulensis*, and *S. chamaecyparissus*, where 0.67, 8.00, and 9.33% seeds germinated, respectively.

The same treatments showed the highest values of mean germination time (MT) as well. On average, pots-treated with EOs of *T. capitata* took 30 days for maximum germination, whereas *E. camaldulensis* and *S. chamaecyparissus* showed a MT of 27.78 and 21.67 days. Although these values were not significantly different from those recorded on the other treatments, all of them were different from both controls.

The values of germination variability coefficient (CVt) showed a high variability in germination time; the calculated value was higher than 100% in all treatments and in the control C2 (water + Fitoil), and showed a lower value (about 45%) only in the water control. No variability coefficient was calculated for the *T. capitata* treatment, since only one germination time value was present.

Mean germination rate (MR) evidenced low values, the minimum being in *T. capitata* and *E. camaldulensis* (0.03 and 0.05, respectively). The two controls C1 and C2 showed the highest values, in both cases slightly higher than 0.1 seeds germinated/day.

The calculated uncertainty of the germination process (U value) was zero for the *T. capitata* treatment, that means no peak of germination occurred; slightly higher values (although not significantly different) could be observed for *S. chamaecyparissus*, *E. camaldulensis*, and *M. piperita* (0.05, 0.07, and 0.09, respectively), whereas a significant difference was observed for the C2 control (water + Fitoil).

Similarly to the variability coefficient (CVt), the synchrony of the germination process (Z index) for the *T. capitata* treatment was not possible to calculate; all the other treatments did not express significant differences. However, a higher degree of overlapping in seeds germination in the *S. chamaecyparissus* treatment is worth noticing. Low synchrony was detectable in the water control (C1), meaning that in that case, seed germination occurred rather gradually.

The lower part of Table 1 shows the effects of the different doses of each tested EO measured during the last survey date (day 40). In all tested EOs, a significant dose–effect occurred, and the inhibitory effect of all EOs was enhanced with increasing doses; at the highest two doses (4 and 8 µL/mL), all EOs had a significantly different effect from the water control (C1). Unlike the other EOs, *T. capitata* exhibited a high effectiveness even at the lowest dose. 

**Figure 1:**
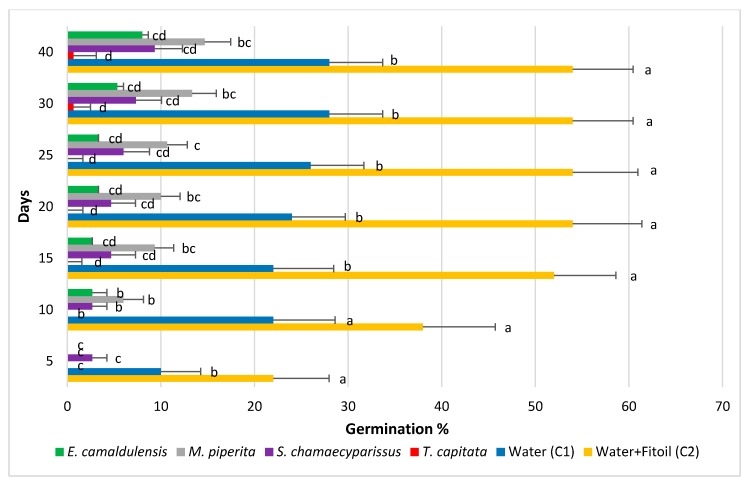
Percentage of seed germination in *Erigeron bonariensis* exposed to four EOs, compared with two controls (C1, water and C2, water + Fitoil). Error bars correspond to the standard error of each mean. For each observation day, means followed by the same letter are not statistically different (Tukey’s test, P ≤ 0.05).

**Figure 2:**
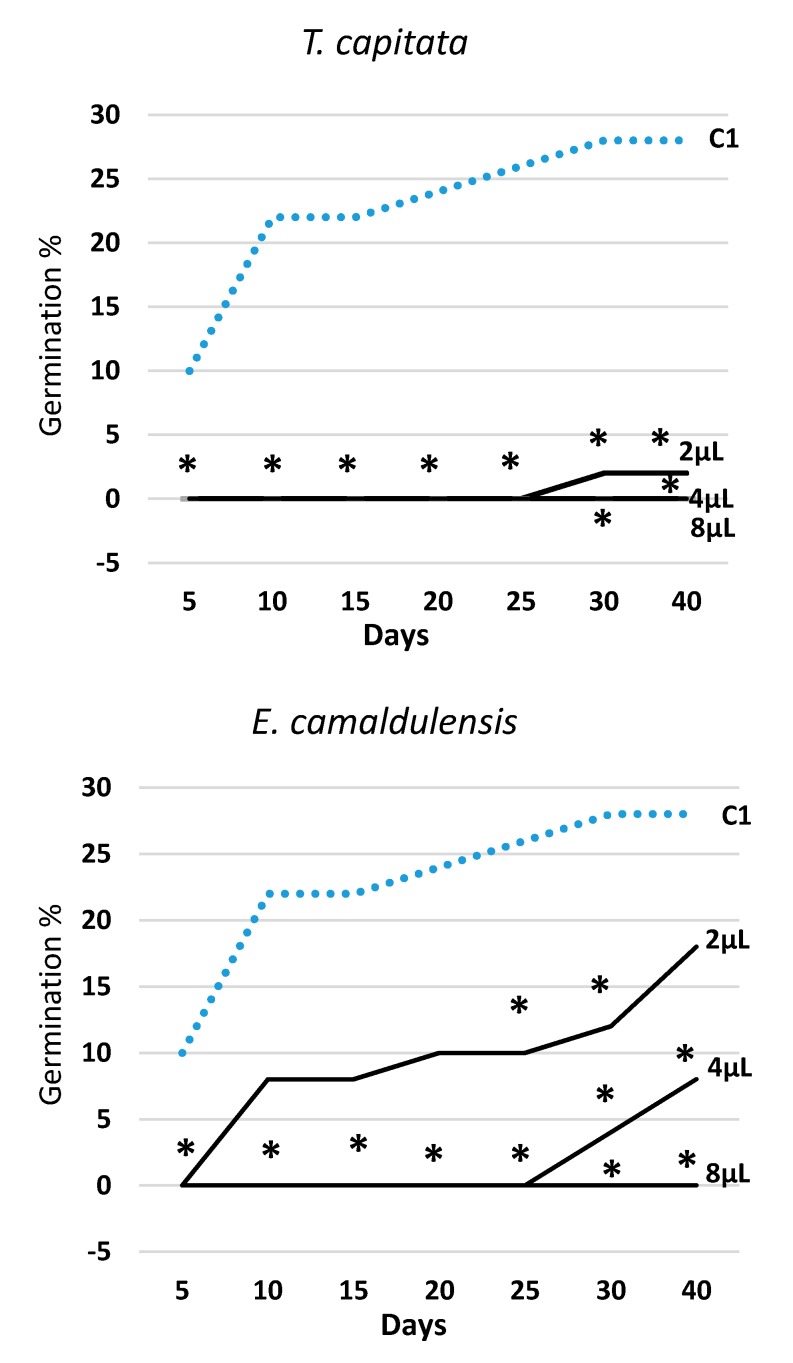

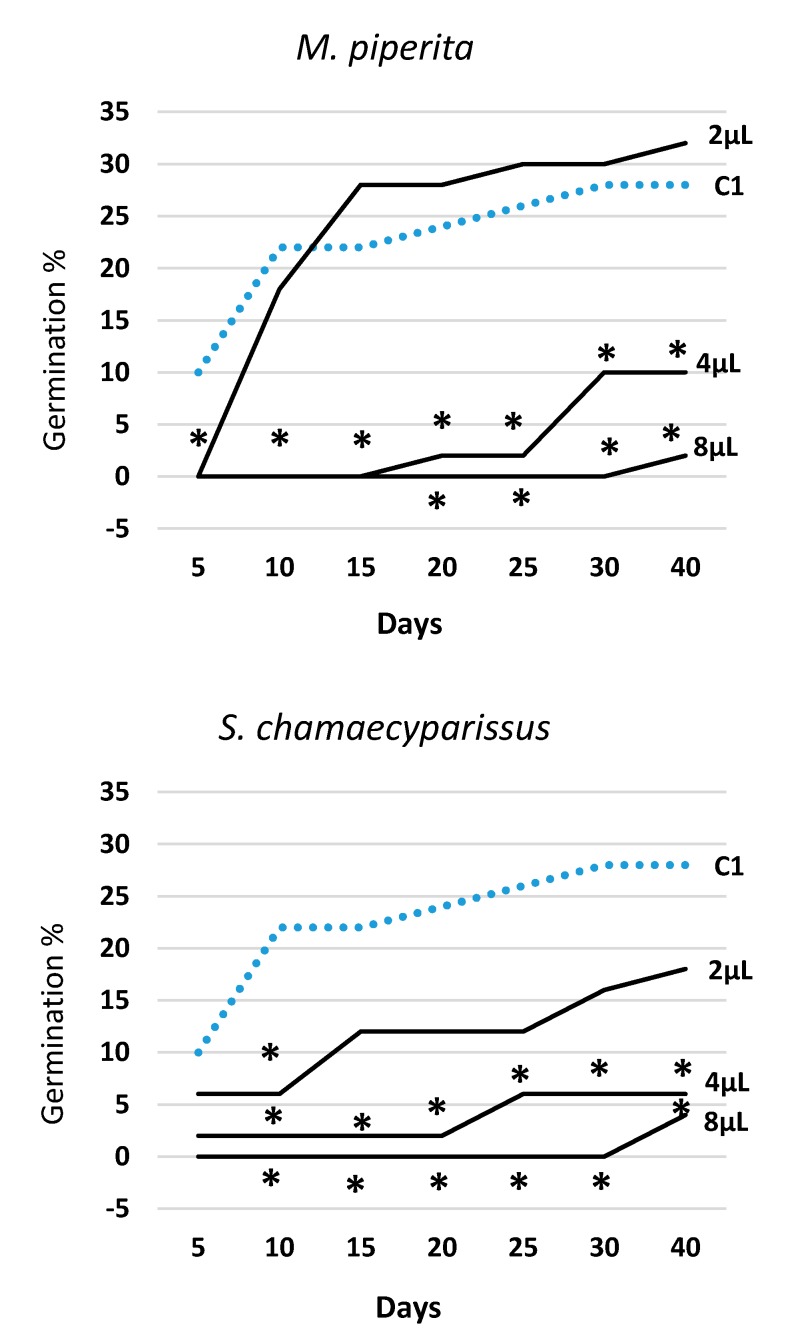
Trend over time of percent seed germination (cumulated values) in *Erigeron bonariensis* exposed to four EOs at three doses (2 μL, 4 μL, 8 μL). For each observation day, values marked with * are significantly different from the water control (C1; dotted line) at P ≤ 0.05 (Dunnett’s test).

Further details on the outcome of the experiment may be retrieved from the data reported in Figure 1 and Figure 2, that show the values of seeds germination (G%) across all survey dates in each EO. Figure 1 shows the values obtained by averaging the administration doses, along with those recorded in the two controls. The highest germination levels were observed in the water + Fitoil control (C2) that kept values higher than all the other treatments, including the water control (C1). The C2 control reached its maximum value (54%) in about 20 days, whereas the water control had a fast increase in the first 10 days, thereafter the germination of new seeds was slower. The germination percentage in the C1 control was rather always about one half than that recorded in the C2. A strong inhibitory effect on seeds germination shows up in the *T. capitata* treatment, where germination values were always lower than 1%. A lower, although remarkable, effect can be seen in the pots treated with *E. camaldulensis* and *S. chamaecyparissus*, where germination reached 8.0 and 9.3%, respectively. Although always lower than in the water control, germination in pots treated with EO of *M. piperita* proved a significant difference with C1 only in few survey dates at ANOVA. In all other treatments, germination was slow and constantly below that measured on the control. 

Figure 2 illustrates the trend over time of germination in all EOs and tested doses (2, 4, and 8 μL) along with the data obtained in the water control (C1). The effectiveness of all EOs varied with the tested dose, always following the rank 2 μL < 4 μL < 8 μL. *T. capitata* EO was effective from the very start of the experiment, and in all survey dates, all three doses were statistically different from the water control (C1). The EOs of *E. camaldulensis* and *S. chamaecyparissus* showed a rather similar effectiveness over time and, when administrated at the lowest dose (2 μL), they—with the exception of few observations—did not behave differently from the water control (C1). Otherwise, the two highest doses (4 μL and 8 μL) allowed a significant reduction of seeds germination in all survey dates. The EO of *M. piperita* expressed a significant effectiveness at the two highest doses as well, but at the lowest dose (2 μL), starting from the third survey date (15 days), it showed even higher germination values than the water control (C1).

### 2.2. Post-Emergence Treatments

Table 2 shows the results of the ANOVA carried out on the values of the phytotoxicity level (PL %) calculated based on the measurements of plants’ diameters that were taken on all survey dates.

As shown, many significant *F* values appear in all first and second order interactions; hence, although some information may be driven by the observation of the mean values, additional analysis was required in order to gain complete information from the data. At first sight, the spraying method would seem more effective than watering, but a low significance level (P < 0.05), together with the occurrence of a highly significant interaction in rather all combinations of factors where M is included, does not allow any generalization. The observation of the “day” (d) factor allows detecting a trend in the expression of phytotoxicity, that appears to be growing until day 15; thereafter, and to the end of the experiment, it stops increasing. The observation of the “treatment” (T) effect allows very well discriminating between the two controls (C1 and C2) by one side, where the presence of high negative values demonstrates a continuous growth of the untreated plantlets, and all treatments, by the other side, that exert, on average, a definite (although variable) inhibitory effect.

**Figure 3:**
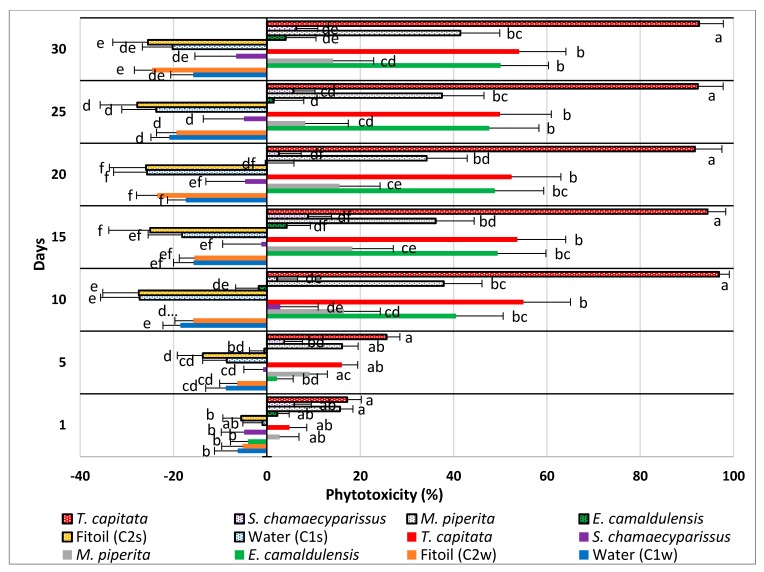
Calculated phytotoxicity level (%) of 4 EOs, distributed by spraying (spotted bars) and watering (full-colored bars) on seedlings of *Erigeron bonariensis*. Error bars indicate the standard error of each mean. For each survey date, values accompanied by the same letter are significantly not different (Tukey’s test, P ≤ 0.05).

The direction and the intensity of this inhibitory effect are illustrated in Figure 3, where the phytotoxicity level of each extract and application method is reported, independently from the used dose, for each survey date.

As shown, all the controls lie on the left side of the graph (beyond the zero axis), showing no phytotoxicity at all; in these cases, the negative values of the PL index throughout the trial even testify an undisturbed growth of plantlets. Overall, the administration by spraying was most effective in the EO of *T. capitata,* whereas watering gave the best results in the EO of *E. camaldulensis;* the other two EOs did not show significant differences between the two supply methods.

The EO of *T. capitata* was the most effective in both administration ways; when sprayed, it reached very quickly (10 days after treatment) a phytotoxicity level close to 100%. When applied by watering, it still kept a phytotoxicity level higher than all other EOs, but the statistical test demonstrates that its efficacy was, on average, similar to that expressed by the EOs of *E. camaldulensis* (watered) and *M. piperita* (sprayed). 

The EO of *E. camaldulensis* was the second most effective EO, but only when administered by watering. This one was the only EO that showed a statistically higher effectiveness when applied by watering; when sprayed, it did not show significant differences from the respective controls (C1s and C2s). 

The EO of *S. chamaecyparissus* showed weak effectiveness only when sprayed, whereas it exhibited no effect at all when watered. In both forms, however, it did not show any statistically significant difference from the controls.

The EOs of *M. piperita* and *S. chamaecyparissus* had statistically the same effectiveness in both application methods. Compared with the respective controls, the first one (*M. piperita*), when sprayed, showed a phytotoxicity level higher than the controls starting from day 10, whereas no differences occurred in the watered treatments. The second EO (*S. chamaecyparissus)* did not show any difference from the controls in both application methods.

**Figure 4:**
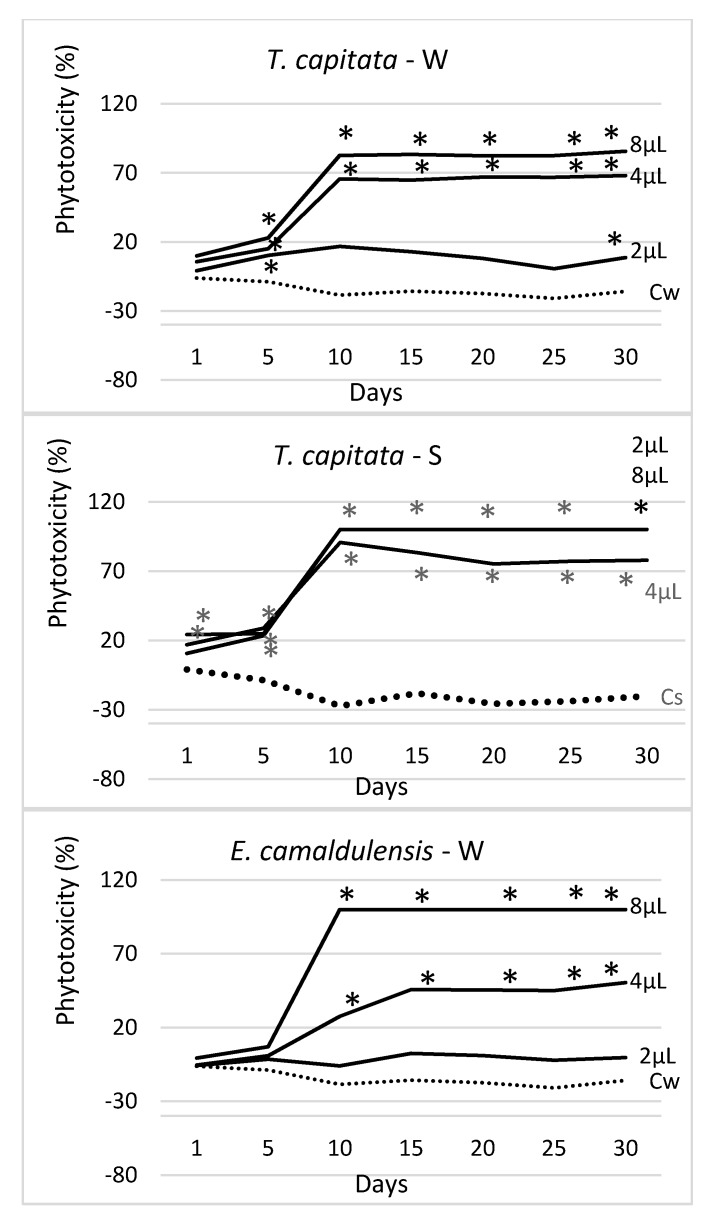

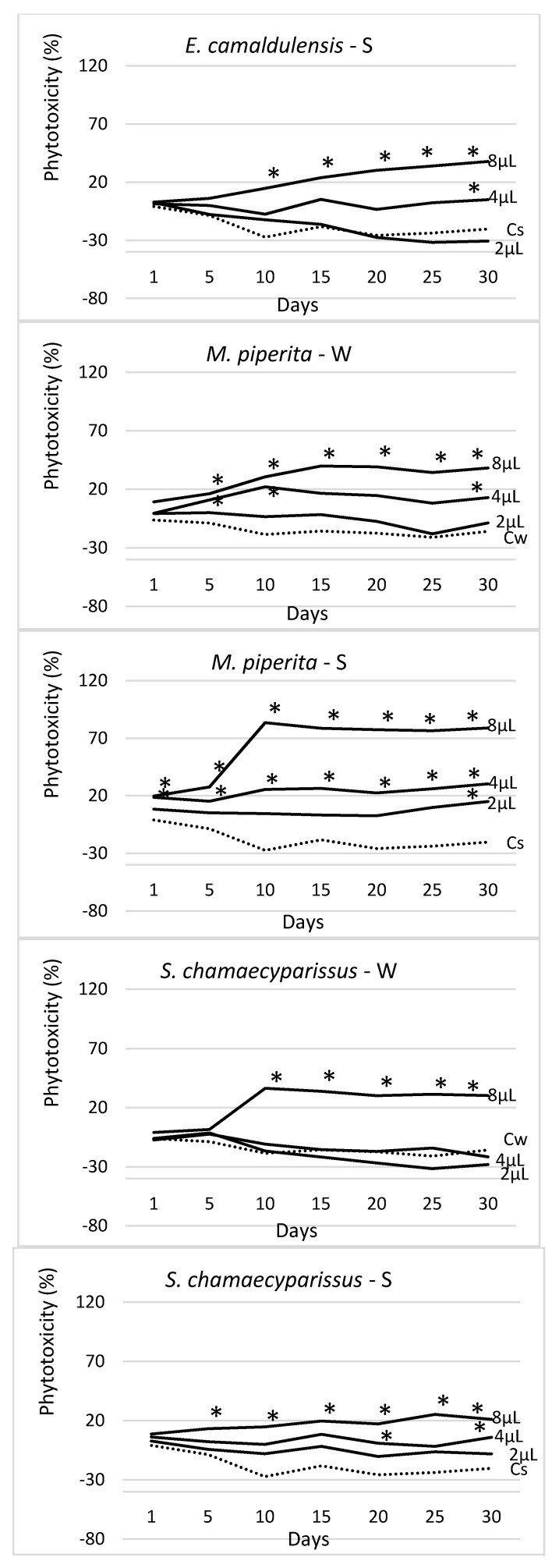
Trend over time of the phytotoxicity level (%) of 4 EOs at three doses (2 μL, 4 μL, 8 μL), distributed by watering (W) and spraying (S) on seedlings of *Erigeron bonariensis*. For each observation day, values marked with * are significantly different from the respective water controls (Cw and Cs; dotted lines) (Dunnett’s test, P ≤ 0.05).

Figure 4 shows the trend over time of the measured phytotoxicity level in each EO, application method, and tested dosage (2 μL, 4 μL, and 8 μL), along with the data obtained in the respective water controls (Cw—watered and Cs—sprayed). In all cases, a strong dose–effect occurred, and the calculated phytotoxicity was growing with the applied dose (2 μL < 4 μL < 8 μL). The EO of *T. capitata* was the most effective of all, especially when sprayed. In this case, at the higher doses (4 μL and 8 μL), it started showing its effectiveness from the very beginning of the experiment (day 1), allowing an effectiveness close to 100% since the 10th day of observation. At the lowest dose (2 μL), this EO proved a higher effectiveness when sprayed. When applied by watering, the phytotoxic effect of *T. capitata* was lower, although, in the last survey, it was, however, significantly different from the control. The *E. camaldulensis* EO was the only one showing a higher effectiveness when administered by watering; at the highest dose (8 μL) it showed complete effectiveness (100% phytotoxicity) since the 10th day of observation, showing no statistical difference with the 8 μL treatment. When sprayed, the *E. camaldulensis* EO showed a lower phytotoxicity level (about 38% at the highest dose), although statistically well differentiated from the water control at the end of the experiment (day 30). The *S. chamaecyparissus* EO was the less effective of all; on day 30 and at 8 μL, it reached a phytotoxicity level of about 30% (with watering distribution) and 21% (after spraying).

### 2.3. Essential Oils Composition

A total of 17 compounds were identified in *T. capitata* EO (Table 3) accounting for 99.80% of the EO composition. This EO was characterized for a high content of carvacrol (72.30%), which was the main compound, followed by *p*-cymene (8.93%) and γ-terpinene (7.77%). In literature, three chemotypes of *T. capitata* have been reported: thymol, carvacrol, and thymol/carvacrol [32,33]. Our sample was carvacrol-type, characterized by high contents of this compound and also by the presence of p-cymene and γ-terpinene in different quantities [32,33,34]. The herbicidal activity of EOs rich in carvacrol from *T. capitata* and other species, as well as of the single compound has been largely studied, although the majority of experiments were performed in in vitro conditions [30,35,36,37,38]. Seeds of *Sinapis arvensis* soaked for 30 min in a solution of 1.5 μL/mL *T. capitata* EO (83.86% carvacrol), dissolved in Tween 20 (0.1%), and then placed on double-layered moistened Whatman No. 1 filter paper in Petri dishes, did not germinate after 10 days of watering [35]. *T. capitata* EO (carvacrol 69.15%) also completely inhibited *S. arvensis* germination and strongly reduced that of *Phalaris canariensis* and *Lolium rigidum* at 1 µL/mL in vitro experiments [29]. In our previous studies, *Erigeron canadensis* and *P. oleracea* germination was blocked at 0.5 and 1 µL/mL by *T. capitata* EO (77.02% carvacrol) in Petri dishes [30]. *E. canadensis* is taxonomically very close to *E. bonariensis,* so we presumed that this EO could exert good herbicidal activity against *E. bonariensis* as well. We also tested pure carvacrol against both weed germinations, preventing it in both species at concentrations starting from 0.0125 µL/mL up to 1 µL/mL, hence we deduced that it was responsible for the herbicidal activity, as suggested by many authors [35,36,37,38,39,40]. It has been suggested that the mode of action of carvacrol as allelochemical is via membrane leakage at high dosages and extensive exposure duration [41].

*M. piperita* EO was constituted by 35 compounds (99.66%) (Table 3), with menthol (51.81%) and menthone (20.52%) being the most abundant, followed by menthyl acetate (6.56%). These are the typical main components of peppermint EO [42,43,44,45,46]. The phytotoxic potential of the EO and water extracts from *M. piperita* is well known [31,47]. The herbicidal activity of *M. piperita* EO has been demonstrated against different weed species *in vivo*, in greenhouse conditions, but it has never been tested on *E. bonariensis*. It was evaluated to control *Amaranthus retroflexus*, *S. arvensis*, and *Lolium* spp. germination at concentrations of 5.4, 21.6, 86.4, and 345.6 mg/L (C1–C4). The C3 dose exerted the maximum inhibitory activity for ryegrass (62%), and wild mustard (44%), while inhibited redroot pigweed 71%. Instead, C4 showed higher inhibition ability only for redroot pigweed (82%), while for wild mustard (32%) and ryegrass (53%) the inhibition was lower than with C3 [48]. A study was conducted to evaluate the stability and other physicochemical properties of some oil-in-water emulsions of *M. piperita* EO with commercially available adjuvants and mixtures with citric or acetic acid, and to determine the short-term phytotoxicity of foliar application of these emulsions and mixtures on *Avena fatua* and *Chenopodium album* in greenhouse experiments [45]. None of the emulsions or mixtures under study exhibited satisfactory stability. The emulsions of *M. piperita* EO had a significant herbicidal effect against *C. album*. None of the adjuvants tested significantly improved the efficacy. The herbicidal effect of emulsions against *A. fatua* depended on the greenhouse temperature, with treatments more effective at higher temperatures. *M. piperita* EO alone induced considerable short-term herbicidal effects. Some adjuvants improved its efficacy shortly after application, but due to regrowth no significant effects on biomass were obtained at harvest. The addition of adjuvants can improve the herbicidal effects of this EO, but further research is necessary [45]. Maffei et al. [46] contributed to determine the mode of action of *M. piperita* EO and its main components, as they were assessed for their ability to interfere with plant plasma membrane potentials. The main component responsible for the depolarization of the membrane was (−)-menthol, followed by (–)-menthone. The results of this study suggested that decreasing water solubility of monoterpenes increases the possibility for terpenoids to interact with and disrupt membrane integrity, thus causing a rapid and irreversible membrane potential depolarization. Since these monoterpenes can cause such extensive membrane depolarization, they are clearly toxic to plant cells. Changes in the bioelectric potential of cells imply changes in the flux of ions across the plasma membrane [46]. 

The EO from *E. camaldulensis* was rich in the oxygenated sesquiterpene spathulenol (31.29%) (Table 3), and also *p*-cymene (20.36%) and cryptone (17%) were important constituents. A total of 25 compounds were identified representing 90.69% of the EO composition. These results are similar to those reported by Verdeguer et al. [14,30]. Furthermore, the EOs from *E. camaldulensis* growing in South Florida, Jerusalem, and Greece were characterized for containing spathulenol, p-cymene, and cryptone as main compounds, and small quantities of 1,8-cineol [49,50,51]. In previous studies, *E. camaldulensis* EO has shown good herbicidal potential, completely blocking the germination of different weed species (*Amaranthus hybridus*, *Portulaca oleracea*, *Conyza canadensis*, and *Parietaria judaica),* and reducing 57% that of *C. album* in in vitro tests [14,30]. The herbicidal activity of *E. camaldulensis* EO rich in 1,8-cineol (32.85%) and p-cymene (23.95%) was tested to control *Convolvulus arvensis*, *Melilotus officinalis*, and *Amaranthus retroflexus* in in vitro conditions. It was ineffective on root and stem growth of *C. arvensis* at 5, 10, and 20 μL concentrations during seven days, while it was effective on root growth of *M. officinalis* and *A. retroflexus* at the same concentrations during seven days [52]. The EO from *E. cladocalyx* cultivated in Morocco, which main compounds were spathulenol (21.6%) and 1,8-cineole (20.5%), followed by p-cymene (15.1%), showed herbicidal activity against *S. arvensis* [53]. To our knowledge, *E. camaldulensis* EO has never been tested against *E. bonariensis* in in vivo conditions so far. Its herbicidal activity could be due to the high content of spathulenol and carvone. The isolated compounds, p-cymene and carvone, and other EO components, were tested against *Lolium rigidum*. Carvone was between the most phytotoxic compounds, completely inhibiting germination and root length of *L. rigidum* at 160 nL/cm^3^ or above, also showing synergistic activity with carvacrol, thymol, and linalool, while *p*-cymene was slightly phytotoxic [38]. However, the herbicidal activity of isolated spathulenol has not been verified yet, because it cannot be purchased and is difficult and expensive to isolate in great quantities as to be tested in in vivo conditions. 

*S. chamaecyparissus* EO possessed the lower content in oxygenated compounds as compared to the other EOs studied (Table 3). Four components were the most abundant: 1,8-cineole (17.50%), viridiflorol (13.56%), germacrene-D (12.60%), and 8-methylene-3-oxatricyclo [5,2,0,0(2,4)] nonane (12.24%). In this EO, 39 compounds were identified (98.95%). The compositions of *S. chamaecyparissus* EOs from different origins have been studied and are very divergent, changing the major compounds and also the number of compounds identified and their abundance [54,55,56,57,58,59]. In some of the studied samples, 1,8-cineole was reported among the most abundant compounds [55,57,59]. The herbicidal effect of *S. chamaecyparissus* EO rich in 1,8-cineole (24.8%) was investigated on seed germination and root and shoot growth of four crops (*Zea mays*, *Triticum durum*, *Pisum sativum*, and *Lactuca sativa*) and two weeds (*Portulaca oleracea* and *Vicia sativa*). It inhibited the germination of both weeds and also of wheat and lettuce and was less harmful for sweet corn and dwarf pea. Regarding the effect on shoot and root length, it was more active on *P. oleracea* than on the crops [57]. The essential oil of *S. chamaecyparissus* from an industrial sample (containing 9.8% of 1,8-cineole and 8.2% of 8-methylene-3-oxatricyclo [5.2.0.02,4] nonane as main compounds) showed a moderate phytotoxicity against the leaf growth of *L. perenne,* but did not show negative effects against *L. sativa* seeds [59]. It has been suggested that *S. chamaecyparissus* EO could be a promising alternative to synthetic herbicides [57,59]. All studies about the herbicidal activity of *S. chamaecyparissus* EO were performed in in vitro conditions. It is the first time that it is tested in vivo and against *E. bonariensis*. As *S. chamaecyparissus* EOs are constituted by a complex mixture of compounds, it has been reported that its activity is due to the synergistic activity of those components [57,58].

## 3. Materials and Methods

### 3.1. Erigeron Bonariensis L. Seeds

The tested seeds were collected from *Erigeron bonariensis* L. plants growing wild in a citrus orchard in the town of Villatorcas (Castellón province, Segorbe, Spain) in July 2016. The botanical identification was performed by the authors using the available specific literature [60]. Before using them in the trials, their germination capability was verified. In vitro germination tests were performed in a germination chamber from Equitec with controlled temperature and light. The chamber conditions were 30 °C 16 h light and 20 °C 8 h dark. Seeds were put in Petri dishes with a diameter of 9 cm (20 seeds for each replication, 5 replications) between two layers of filter paper (73 g·m^−2^) and wetted with 5 mL of distilled water to germinate. Petri dishes were sealed with Parafilm^®^ and placed in the germination chamber for 15 days. Seeds germinated with percentages of around 75%.

### 3.2. Essential Oils (EOs)

To test their herbicidal activity against *E. bonariensis*, *Thymbra capitata* (L.) Cav., *Mentha piperita* L., and *Santolina chamaecyparissus* L. EOs were purchased, respectively, from Bordas S.A., Sigma-Aldrich and Ecoaromuz. *Eucalyptus camaldulensis* Dehnh. EO is not commercially available and was obtained by hydrodistillation from fresh leaves collected from trees growing as ornamentals in the Turia River Gardens of Valencia, Spain.

To extract the oil, two Clevenger apparatus were used together with round-bottom flasks of 2 and 4 L, heating mantles, and a condenser. After the fresh material was weighted with a precision scale, it was introduced in the flasks and 1000 mL (for the 2 L flask) or 2000 mL (for the 4 L flask) of distilled water were added. Heat was applied to the round-bottom flask by the heating mantle in order to generate water vapor carrying the volatile compounds of the drug. Then it was cooled in the condenser and passed to the graduated collector tube, where the essential oil was separated from the water. This process was carried out for at least 3 h, ending when no more amount of oil was obtained for 30 min. The yield of extraction was about 1.7 mL of EO per 100 g of leaves.

All EOs were kept in the refrigerator at 4 °C until they were used.

### 3.3. Gas Chromatography (GC)

The quantification of the EOs constituents was performed by gas chromatography using a Clarus 500GC Perkin–Elmer apparatus equipped with a flame ionization detector (FID), and a capillary column ZB-5 (30 m × 0.25 mm i.d. × 0.25 μm film thickness). The injection volume was 1 μL. The GC oven temperature was set at 60 °C for 5 min, with 3 °C increases per min to 180 °C, then 20 °C increases per min to 280 °C, which was maintained for 10 min. Helium was the carrier gas (1.2 mL/min). Injector and detector temperatures were set at 250 °C. The percentage composition of the EO was computed from GC peak areas without correction factors by means of the software Total Chrom 6.2 (Perkin-Elmer Inc., Wellesley, PA, USA).

### 3.4. Gas Chromatography−Mass Spectrometry (GC–MS)

For the identification of the compounds, gas chromatography coupled to mass spectrometry (GC–MS) was performed using a Clarus 500 GC–MS from Perkin-Elmer Inc. apparatus equipped with the same capillary column, carrier, and operating conditions as described above for GC analysis. The ionization source temperature was set at 200 °C and an electron impact mode of 70 eV was employed. MS spectra were obtained by means of total ion scan (TIC) mode (mass range *m*/*z* 45–500 uma). The total ion chromatograms and mass spectra were processed with the Turbomass 5.4 software (Perkin-Elmer Inc.). Retention indexes were determined by injection of C_8_–C_32_ n-alkanes standard under the same conditions.

The EO components were identified by comparison of their mass spectra with those of the computer library NIST MS Search 2.0 and available data in the literature [61]. Identification of the following compounds was confirmed by comparison of their experimental RI with those of authentic reference standards (Sigma-Aldrich, Darmstadt, Germany): α-pinene, β-pinene, camphene, myrcene, limonene, (*Z*)-β-ocimene, camphor, borneol, terpinen-4-ol, bornyl acetate, and linalool.

### 3.5. Greenhouse Conditions

All tests were conducted from 19 May to 24 August, 2017; the average temperature of the greenhouse was 27.3 °C with a maximum temperature of 36.5 °C (August) and a minimum of 19.1 °C (July). The relative humidity was between 24.8 and 93.4% (Table 4).

### 3.6. Pot Preparation

For all experiments, pots of 8 × 8 × 7 cm were used, filled with 200 g of a substrate mixture of ¾ of peat moss and ¼ of perlite, previously homogenized.

### 3.7. Pre-Emergence Herbicidal Tests

Pots were irrigated with 2/3 of their water holding capacity (WHC), 60 mL. Five seeds of *E. bonariensis* were placed in each pot, and 10 replicates were performed for each treatment, including controls. Treatments consisted of 4 EOs (*T. capitata*, *M. piperita*, *E. camaldulensis*, and *S. chamaecyparissus*), each of them distributed at three doses (2, 4, and 8 µL/mL), plus one control with water (C1) and one control with water + Fitoil at 1 µL/mL (C2). Fitoil is a biological adjuvant with 40% soybean oil, used to emulsify the EOs with water, supplied by Xeda Italia s.r.l. After sowing, the remaining 1/3 of the WHC, 30 mL for each treatment was added to the corresponding pots.

During the experiment, which lasted 30 days, pots were irrigated three times per week.

The effects of the treatments on seed germination were tested by means of the germination measurements suggested by [62,63]. Pots were checked on a daily basis, and seeds were considered fully germinated after the full spreading of cotiledonary leaves. The daily count of every newly-germinated seed was used for determining the value of seed germination, further expressed as percentage (G%). Mean germination time (MT), expressed in days, indicates the average length of time required for maximum germination of the tested seeds lot, and was calculated according to the following formula:

(1) $$\mathrm{MT}\;=\;\frac{\sum_{i=1}^{k}n_{i}t_{i}}{\sum_{i=1}^{k}n_{i}},$$

where:

*t_i_* = days from the start of the experiment to the ith observation;

*n_i_* = number of seeds newly germinated in the ith day;

k = last day of germination.

The coefficient of variation of the germination time (CVt) expressed in percentage was obtained as follows:

CVt = 100 st/MT,(2)

where:

st = standard deviation of the germination time;

MT = mean germination time.

MR (mean germination rate) is the average number of seeds germinated in the time unit (in our case, one day), and is calculated as the reciprocal of MT.

The synchrony of the germination process was evaluated by means of the indices U and Z.

U is the uncertainty of the germination process, i.e., the uncertainty associated with the distribution of the relative frequency of germination (*f*_i_). It is expressed in bit (binary measurement), since it is based on the counts of the condition germinated/not germinated. It can assume values spanning from 0 and $\log_{2}$n, with n being the total number of seeds germinated; low values of U indicate frequencies with few peaks, i.e., a germination process that was more concentrated in time. The index U is calculated through the following formula:

(3) $$U=\;-\;\sum_{i=1}^{k}f_{i}\log_{2}f_{i},$$

where *f*_i_ is the relative frequency of germination, on its turn calculated as

(4) $$f_{i}\;=\;n_{i}/\sum_{i=1}^{k}n_{i}.$$

Z is the synchrony of the germination process, i.e., a measurement of the degree of overlapping of seeds germination in time. It can take values from 0 (when at least two seeds germinated, in two different moments) to 1 (when all seeds germinated at the same time). It is calculated with the following formula:

(5) $$Z\;=\;\sum^{\;}C_{ni,\;2}/N,$$

where $\;C_{ni,\;2}$ is the combination of the seeds, two together, germinated in the time i, and N is the sum of such combinations, along the whole experiment:

(6) $$N\;=\;\sum^{\;}n_{i}(\sum^{\;}n_{i}-1)/2.$$

All data were submitted to a two-way ANOVA, setting the six treatments (T) as independent variables, including the controls and the EO doses (D) within each treatment. When the *F* test gave a significant response, the differences among T values were appreciated through the Tukey’s test, whereas each dose within treatment (D × T) value was compared with the respective value of the water control C1 (Dunnett’s test) [64].

Percentage values were transformed into angular values, according to the following formula:

(7) $$Y=\arcsin\;\sqrt{p}$$

where:

Y = transformed value;

p = percentage.

Before transformation, all zero values were substituted by (1/4n), where n was the number of units upon which the percentage data was based (in our case, n = 10) [64]. The data reported in the table, however, are the original values.

### 3.8. Post-Emergence Herbicidal Test

The same treatments tested in pre-emergence were tested in post-emergence, using two different ways of applying them: watering or spraying in order to determine the best way to maximize the herbicidal activity of EOs. Each treatment was applied on *E. bonariensis* plants at the rosette phenological stage, that were previously produced from the same lot of seeds used for the pre-emergence trials. Ten repetitions (ten pots with one plant per pot) were used for each treatment. For both applications, 20 mL of each EO and dose were applied in the corresponding pots. The spraying was carried out with a manual trigger sprayer.

The first evaluation was made 24 h after treatment. Then, evaluations were carried out every five days, taking images of the pots that were later processed with the software Digimizer v.4.6.1 (MedCalc Software, Ostend, Belgium, 2005–2016). During the experiment, which lasted 30 days, pots were irrigated three times per week.

The diameter (cm) of each plantlet was measured 1, 5, 10, 15, 20, 25, and 30 days after treatments application, including the controls with water and water + Fitoil, termed Cw and Cf, respectively. The phytotoxicity level (PL) of each treatment was assessed by determining the percent variations of growth of plantlets, both in treatments and in the controls, 1, 5, 10, 15, 20, 25, and 30 days after treatment, according to the following formula:

PL = (l_t1_ − l_t2_)/l_t1_ × 100, (8)

where:

l_t1_ = diameter (cm) of plantlet at day t_1_;

l_t2_ = diameter (cm) of plantlet at day t_2_.

As such, a PL = 100 means complete inhibition effect (wilted plantlet), whereas a PL < 1 means stimulation effect (growing plantlet, i.e., unaffected by the treatment).

All data were submitted to a multi-way ANOVA, setting as independent variables the method of application (M: watering or spraying), the day of survey (d: 1, 5, 10, 15, 20, 25, and 30 days after the treatment), the six treatments (T), including the controls and the EO doses within each treatment. The effect of the three doses was checked against the respective water control (Cw) by means of the Dunnett’s test, and this analysis was repeated separately for all survey dates and methods of application.

## 4. Conclusions

All the EOs tested significantly reduced *E. bonariensis* seed germination, ranking *T. capitata* > *E. camaldulensis* > *S. chamaecyparissus* > *M. piperita*. In post-emergence assays, *T. capitata* EO was the most effective in both administration ways, acting quicker when sprayed. *E. camaldulensis* EO was the second most effective but only when administrated by watering. The EOs of *T. capitata*, *M. piperita*, and *E. camaldulensis* demonstrated a high potential to control *E. bonariensis* and could be a source for the development of natural herbicides. It is important to highlight that the herbicidal activity of the EOs tested depended on the way they were administrated. It is necessary to develop adequate formulations to enhance the activity of the EOs and try them in field conditions.

## Figures and Tables

**Table 1:** Germination measurements (mean ± standard deviation) and results of ANOVA of seeds of *Erigeron bonariensis* treated in pre-sowing with essential oils (EOs) of *Eucalyptus camaldulensis, Mentha piperita, Santolina chamaecyparissus*, and *Thymbra capitata* at different doses. Treatment means followed by the same letters in each column are not significantly different at P ≤ 0.05 (Tukey’s test). Within each DxT group, values not followed by the superscript “a” are not significantly different from the water control (C1) at P ≤ 0.05 (Dunnett’s test). Prior to analyses, percent values were transformed into angular values.

		G (%)			MT (days)			CVt (%)			MR (days^−1^)			U (bit)			Z		
		DF	Mean ± st.dev.		DF	Mean ± st.dev.		DF	Mean ± st.dev.		DF	Mean ± st.dev.		DF	Mean ± st.dev.		DF	Mean ± st.dev.	
Treatment (T)		5			5			5			5			5			4		
	Water (C1)		28.00 ± 21.50	B		13.23 ± 9.07	C		42.10 ± 35.75	B		0.1033 ± 0.0527	AB		0.28 ± 0.46	AB		0.00 ± 0.00	
	Water + Fitoil (C2)		54.00 ± 18.97	A		9.96 ± 2.82	B		135.50 ± 16.24	A		0.1097 ± 0.0387	A		0.56 ± 0.49	A		0.50 ± 0.41	
	*E. camaldulensis*		8.00 ± 13.49	CD		27.78 ± 12.28	A		149.73 ± 13.17	A		0.0474 ± 0.0309	B		0.07 ± 0.25	B		0.33 ± 0.58	
	*M. piperita*		14.67 ± 16.55	BC		19.32 ± 10.14	A		139.95 ± 5.01	A		0.0662 ± 0.0307	AB		0.09 ± 0.34	B		0.60 ± 0.55	
	*S. chamaecyparissus*		9.33 ± 15.52	CD		21.67 ± 12.62	A		142.24 ± 1.42	A		0.0767 ± 0.0713	AB		0.05 ± 0.29	B		0.67 ± 0.58	
	*T. capitata*		0.67 ± 3.65	D		30.00 ± 00.00	A					0.0333 ± 0.0000	AB		0.00 ± 0.00	B			
			*F*(5,126) = 27.82 ***		*F*(5,43) = 8.46 ***			*F*(5,18) = 22.62 ***			*F*(5,42) = 3.53 **			*F*(5,126) = 6.80 ***			*F*(4,18) = 1.49 n.s.		
Dose (D) within treatment (T)		8			5			1			5			8			1		
*E. camaldulensis*	2 µL		18.00 ± 17.51			23.33 ± 12.52			149.73 ^a^ ± 13.17			0.0573 ± 0.0343			0.20 ± 0.42			0.33 ± 0.58	
	4 µL		6.00 ^a^ ± 9.66			36.67 ^a^ ± 5.77						0.0278 ^a^ ± 0.0048			0.00 ± 0.00				
	8 µL		0.00 ^a^ ± 0.00												0.00 ± 0.00				
*M. piperita*	2 µL		32.00 ± 13.98			12.92 ± 5.26			139.95 ± 5.01			0.0851 ± 0.0219			0.26 ± 0.56			0.60 ± 0.55	
	4 µL		10.00 ^a^ ± 10.54			28.00 ^a^ ± 4.47						0.0367 ^a^ ± 0.0075			0.00 ± 0.00				
	8 µL		2.00 ^a^ ± 6.32			40.00 ^a^ ± 0.00						0.0250 ± 0.0000			0.00 ± 0.00				
*S. chamaecyparissus*	2 µL		18.00 ± 19.89			20.28 ± 12.54			142.65 ^a^ ± 1.73			0.0770 ± 0.0709			0.16 ± 0.50			0.50 ± 0.71	
	4 µL		6.00 ^a^ ± 9.66			18.33 ± 11.55						0.0933 ± 0.0924			0.00 ± 0.00				
	8 µL		4.00 ^a^ ± 12.65			40.00 ± 0.00			141.42 ± 0.00			0.0250 ± 0.0000			0.00 ± 0.00			1.00 ± 0.00	
*T. capitata*	2 µL		2.00 ^a^ ± 6.32			30.00 ± 0.00						0.0333 ± 0.0000			0.00 ^a^ ± 0.00				
	4 µL		0.00 ^a^ ± 0.00												0.00 ^a^ ± 0.00				
	8 µL		0.00 ^a^ ± 0.00												0.00 ^a^ ± 0.00				
			*F*(8,126) = 7.33 ***			*F*(5,43) = 5.83 ***			*F*(1,18) < 1 n.s.			*F*(5,42) = 1.55 n.s.			*F*(8,126) = 1.29 n.s.			*F*(1,18) < 1 n.s.	
Error		126			43			18			42			126			18		
Total		139			53			23			52			139			23		

G, seed germination (%); MT, mean germination time (days); CVt, germination variability coefficient (%); MR, mean germination rate (days^-1^); U, uncertainty of the germination process (bit); Z, synchrony of the germination process (adimensional). Significance of *F* values: ** 0.001 < P ≤ 0.01; *** P ≤ 0.001; n.s., not significant.

**Table 2:** Phytotoxicity level (mean ± standard deviation of main effects) and results of ANOVA on *Erigeron bonariensis* plantlets exposed to four EOs, applied by watering or spraying, at different doses and evaluated at 1, 5, 10, 15, 20, 25, and 30 days after treatment.

		Phytotoxicity	
Source		DF	Mean ± st. dev.
	Method of application (M)	1	
	Watering		11.85 ± 47.38
	Spraying		15.81 ± 46.41
			*F* (1, 2148) = 4.73 *
	day (d)	6	
	1		2.59 ± 21.07
	5		4.33 ± 22.52
	10		17.87 ± 51.99
	15		20.08 ± 52.26
	20		16.76 ± 53.92
	25		16.56 ± 54.66
	30		18.62 ± 53.54
			*F* (6, 2148) = 9.33 ***
	Treatment (T)	5	
	Water (C1)		−16.28 ± 25.75
	Water + Fitoil (C2)		−18.66 ± 26.76
	*E. camaldulensis*		17.42 ± 45.48
	*M. piperita*		21.67 ± 42.36
	*S. chamaecyparissus*		1.08 ± 33.84
	*T. capitata*		56.9 ± 49.34
			*F* (5, 2148) = 271.91 ***
	Dose within treatment (T)	8	*F* (8, 2148) = 61.2 ***
	M*d	6	*F* (6, 2148) < 1 n.s.
	M*T	5	*F* (5, 2148) = 48.87 ***
	d*T	30	*F* (30, 2148) = 9.19 ***
	M*d*T	30	*F* (30, 2148) = 2.38 ***
Error		2148	
Total		2239	

Significance of *F* values: * 0.01 < P ≤ 0.05; ** 0.001 < P ≤ 0.01; *** P ≤ 0.001; n.s., not significant significant.

**Table 3:** Chemical composition of *Thymbra capitata* (TC), *Mentha piperita* (MP), *Eucalyptus camaldulensis* (EC), and *Santolina chamaecyparissus* (SC) EOs.

Component	KI_._	TC	MP	EC	SC
Monoterpene hydrocarbons		22.54	1.95	22.27	9.30
Santolina triene	908	-	-	-	0.13
α-Thujene	930	0.89	0.01	0.43	-
α-Pinene	938	0.74	0.28	-	0.85
Thuja-2,4(10)-diene	947	-	-	0.10	-
Camphene	951	-	-	-	0.28
Sabinene	975	-	0.14	0.09	0.17
β-Pinene	978	0.29	0.43	-	3.98
Myrcene	991	1.95	0.01	0.08	-
α-Phellandrene	1004	0.16	-	0.11	-
γ-Terpinene	1016	**7.77**	0.13	0.12	1.18
α-Terpinene	1016	1.61	-	-	0.69
*p*-Cymene	1025	**8.93**	0.18	**20.36**	2.01
Limonene	1029	0.20	0.73	0.87	-
(Z)-β-Ocimene	1040	-	0.03	-	-
*iso*-Terpinolene	1087	-	0.02	-	-
*p*-Cymenene	1090	-	-	0.11	-
Oxygenated monoterpens		73.98	95.35	33.76	39.32
1,8-Cineole	1031	0.11	4.31	2.31	**17.50**
*trans*-Pinocarveol	1037	-	-	-	0.17
Artemisia ketone	1062	-	-	-	4.63
(Z)-Sabinene hydrate	1070	-	0.76	-	-
Linalool	1097	0.77	0.09	-	0.42
*trans*-Thujone	1117	-	-	0.19	-
Camphor	1142	-	-	-	4.03
Menthone	1154	-	**20.52**	-	-
(E)-Pinocamphone	1159	-	-	-	0.18
(Z)-Chrysanthemol	1162	-	-	-	3.80
Menthofuran	1163	-	5.21	-	-
*neo*-Menthol	1165	-	3.12	-	-
Borneol	1168	0.16	-	-	1.11
(Z)-Pinocamphone	1172	-	-	-	2.03
Menthol	1175	-	**51.81**	-	-
Terpinen-4-ol	1177	0.37	0.67	2.89	2.69
*iso*-menthol	1182	-	0.60	-	-
Neoisomenthol	1187	-	0.08	-	-
α-Terpineol	1188	-	0.17	0.93	0.21
Myrtenal	1192	-	-	-	1.31
Myrtenol	1193	-	-	-	1.07
Cryptone	1196	-	-	**17.00**	-
Verbenone	1198	-	-	-	0.16
*m*-Cumenol	1230	-	-	0.46	-
Pulegone	1236	-	0.83	-	-
Cumin aldehyde	1245		-	4.15	-
Carvotanacetone	1250	-	-	0.33	-
Piperitone	1251	-	0.32	-	-
*neo*-Menthyl acetate	1273	-	0.16	-	-
*p*-Menth-1-en-7-al	1279	-	-	2.85	-
Menthyl acetate	1291	-	**6.56**	-	-
Thymol	1292	0.27	-	1.14	-
Carvacrol	1300	**72.30**	-	1.51	-
*iso*-Menthyl acetate	1303	-	0.16	-	-
Sesquiterpene hydrocarbons		3.14	2.22	0.19	21.78
α-Ylangene	1373	-	-	-	0.08
α-Bourbonene	1381	-	0.17	-	-
β-Caryophyllene	1415	3.14	1.47	-	0.39
β-Farnesene	1454	-	0.02	-	-
*allo*-Aromadendrene	1457	-	-	0.19	4.23
*trans*-Cadina-1(6),4-diene	1473				0.36
Germacrene-D	1477	-	0.42	-	**12.60**
β-Selinene	1491	-	0.13	-	-
Elixene	1492	-	-	-	2.80
γ-Cadinene	1509	-	-	-	0.32
δ-Cadinene	1519	-	-	-	1.00
Oxygenated sesquiterpenes		0.14	0.00	34.47	15.64
Bornyl acetate	1283	-	-	-	0.08
Spathulenol	1477	-	-	**31.29**	1.42
Caryophyllene oxide	1577	0.14	-	-	0.19
Viridiflorol	1587	-	-	0.54	**13.56**
β-Oplopenone	1602	-	-	-	0.16
Spathulenol isomer	1616	-	-	1.29	-
*iso*-spathulenol	1640	-	-	1.35	-
α-Cadinol	1649	-	-	-	0.23
Others		0.00	0.14	0.00	12.91
1-Butanol, 2-methyl-, propanoate	973	-	-	-	0.20
1-Octen-3-ol	980	-	0.02	-	-
3-Octanol	995	-	0.07	-	-
*iso*-Amyl 2-methyl butyrate	1101	-	0.02	-	-
*n*-Amyl isovalerate	1106	-	0.04	-	0.48
8-methylene-3-oxatricyclo[5,2,0,0(2,4)]nonane	1117	-	-	-	**12.24**
TOTAL IDENTIFIED (%)		99.80	99.66	90.69	98.95

In bold, the most significant compounds for each EO.

**Table 4:** Greenhouse temperature and relative humidity conditions during the experimental period.

Month	Temperature (°C)			Relative Humidity (R.H.) %		
	Mean	Maximum	Minimum	Mean	Maximum	Minimum
May	25.0	33.2	19.2	64.5	81.2	32.3
June	27.9	36.3	20.1	61.4	82.1	24.8
July	27.9	35.6	19.1	69.0	93.4	36.8
August	28.4	36.5	22.3	68.4	86.7	42.8
